# Trends in Testing for SARS-CoV-2 Among Healthcare Workers in a Canadian Cohort Study During the COVID-19 Pandemic, June 2020 to November 2023

**DOI:** 10.1155/cjid/1858884

**Published:** 2025-05-30

**Authors:** Nicole M. Robertson, Brenda L. Coleman, Robyn Harrison, Curtis Cooper, Jeya Nadarajah, Marek Smieja, Jeff Powis, CCS Working Group

**Affiliations:** ^1^Sinai Health, 600 University Ave, Toronto M5G 1X5, Ontario, Canada; ^2^School of Public Health, University of Toronto, 155 College St, Toronto M5T 3M7, Ontario, Canada; ^3^Division of Infectious Diseases, University of Alberta, 8440 112 St, Edmonton T5J 3E4, Alberta, Canada; ^4^Faculty of Medicine, University of Ottawa, Roger Guindon Hall, 451 Smyth Rd #2044, Ottawa K1H 8M5, Ontario, Canada; ^5^Oak Valley Health, 381 Church St, Markham L3P 13P, Ontario, Canada; ^6^St. Joseph's Healthcare, 50 Charlton Ave East, Hamilton L8N 4A6, Ontario, Canada; ^7^Michael Garron Hospital, 825 Coxwell Avenue, Toronto M4C 3E7, Ontario, Canada

## Abstract

**Background:** While testing healthcare workers (HCWs) for SARS-CoV-2 is important to reduce transmission within healthcare settings, understanding the self-reported patterns of testing is important for interpreting vaccine effectiveness and other COVID-19-related information.

**Objective:** Using longitudinal data from the COVID-19 cohort study, this study described trends in SARS-CoV-2 testing among Canadian HCWs between June 2020 and November 2023.

**Methods:** HCWs completed an illness report for each instance of SARS-CoV-2 testing and episodes of symptoms compatible with COVID-19 even if untested. Overall rates of testing among the participating cohort were calculated. Rates were stratified by province, reason for testing and COVID-19 vaccination status using 4-week intervals to smooth estimates. For episodes of symptomatic illness (only), the median time between symptom onset and first test was calculated, along with the percent of episodes initially receiving a negative result for SARS-CoV-2 that were reported as being retested.

**Results:** Rates of testing for SARS-CoV-2 generally mirrored rates of hospitalisation for COVID-19 among Canadians. Rates of testing were highest during the Omicron BA.1 wave (11.9 participants tested at least once per 1000 person-days) and varied by province; vaccination status did not impact rates. The most common reason for testing was for symptoms. Testing for known exposure or routine reasons greatly decreased after the Omicron BA.1 wave. In participants who were tested for episodes of symptomatic illness, the median time between symptom onset and first test was 1 day (interquartile range 0–2). Reported retesting after an initial negative result remained low throughout the study period.

**Conclusions:** Understanding testing behaviours is important for public health decision-making including the analysis and interpretation of case data and vaccine effectiveness studies. It can also highlight possible missed case–finding opportunities in healthcare settings.

## 1. Introduction

Diagnostic testing for agents of infectious diseases such as severe acute respiratory syndrome coronavirus-2 (SARS-CoV-2) is imperative to facilitate timely treatment to improve prognosis and to trigger transmission-limiting behaviours [1, 2]. At the facility level, the suspicion or confirmation of an infectious patient or healthcare worker (HCW) should prompt the implementation of enhanced infection prevention and control measures intended to protect other patients and staff [3]. At the population level, testing is a key element in population-based surveillance to inform public health intervention planning [4]. For researchers, participant-level self-reports represent a useful method that can enhance formal reporting methods in the interpretation of findings [5].

There have been numerous changes in testing methods, access to tests, and in attitudes and behaviours for SARS-CoV-2 testing in Canada. Eligibility and testing criteria, availability of tests, type of tests and reporting requirements have all changed over time [6]. At the beginning of the coronavirus disease 2019 (COVID-19) pandemic, testing was primarily completed using polymerase chain reaction (PCR) tests that were administered in designated facilities by trained individuals and processed in the public health laboratory systems [7]. Testing criteria were narrow and largely focused on individuals with symptoms compatible with COVID-19, a history of travel or known exposure to a confirmed case of COVID-19 [6]. By May 2020, supply chain issues eased and testing was expanded [7]. By early 2022, rapid antigen tests (RATs) were widely deployed across Canada to enhance testing capacity [8]. PCR tests were still available and recommended for people at a high risk of serious outcomes and for those working in high-risk institutions, such as acute care facilities [9–12]. While there are benefits to increased availability and accessibility to testing, one drawback of RATs is the decoupling from the public health reporting systems that then hinders surveillance measures [13].

Observational study designs are commonly employed to evaluate real-world vaccine effectiveness [14]. As such, it is important to understand differences that may bias the results. Beyond differences in barriers to testing and test accuracy, differences may occur in test-seeking behaviour, such as between vaccinated and unvaccinated individuals [15–17]. It is therefore important to understand the behaviours that lead to testing and to assess why and how often individuals seek testing [18].

Studies investigating SARS-CoV-2 testing trends among underserved or avoidant populations in Canada found lower rates of testing among individuals who were identified as male [19–23] and had lower levels of education [21, 23] and who lived in rural areas [19] or areas with higher densities of visible minority populations [23]. Conversely, higher rates of testing were noted in people with a known exposure to COVID-19 [20] or who had symptoms of COVID-19 [21]. Several studies noted variations or discrepant results in the effects of age, household income and other social determinants of health, as well as between Canadian provinces [19–23]. The differences between provinces may reflect the influences of time and evolving provincial policies on testing behaviours. To the best of these authors' knowledge, no studies have investigated the trends in SARS-CoV-2 testing in Canadian HCWs.

The COVID-19 Cohort Study (CCS) collected data from HCWs between June 2020 and November 2023, a period of many COVID-19–related scientific advances (e.g., vaccine development and deployment) and policy and attitude changes [24]. These data provide a unique opportunity to analyse testing patterns that would not be evident through traditional public health reporting.

This study sought to describe trends in SARS-CoV-2 testing among Canadian HCWs enrolled in the CCS from June 2020 to November 2023. Specifically, the objective was to describe temporal trends in rates of participant testing across geographic regions, vaccination status and reasons for testing as well as to describe testing patterns within episodes of illnesses with COVID-19 compatible symptoms.

## 2. Materials and Methods

### 2.1. Design and Participants

This study was conducted as part of the CCS, a 42-month prospective cohort study following a group of HCWs working in acute care, rehabilitation and complex care hospitals (facilities for patients with complex medical conditions that no longer require acute care but require care above what can be provided at home or a long-term care facility) [25], across four Canadian provinces [26]. Recruitment occurred immediately following ethical approval at each site, with staggered enrolment from June 2020 to June 2023. Recruitment commenced in Ontario in June 2020, followed by Alberta in March 2021, Nova Scotia in May 2021 and Quebec in June 2021 (see Supporting Figure 1). Recruitment, consent and data collection were conducted electronically due to COVID-19 restrictions on in-person research activities. Data were collected anonymously using a secure bespoke online platform; collection ended on 1 December 2023, or upon participant withdrawal, whichever occurred first.

HCWs were eligible for inclusion in these analyses if they were 18–75 years of age at the time of enrolment and (a) employed > 20 h per week by an acute care, rehabilitation or complex care hospital; or (b) were a physician or nurse practitioner with hospital privileges and caring for ill patients ≥ 8 h per week or (c) worked in a medical office providing patient care for > 20 h per week. Eligibility was restricted to participants who participated in the study for ≥ 30 days and completed at least one baseline survey. Participants were eligible irrespective of their COVID-19 vaccination status or history of COVID-19.

### 2.2. Testing for SARS-CoV-2

As active case identification was imperative for many of the proposed analyses of the CCS, efforts were made to ensure participants had access to SARS-CoV-2 testing throughout the study. At the initiation of the study, PCR testing was available to both HCWs and the public [7]. RAT kits became publicly available on December 2021 in Quebec, Nova Scotia and Alberta [27–29] and by February 2022, in Ontario [30]. As of 1 April 2023, COVID-19 assessment centres in most provinces closed and RATs were no longer being distributed but were available as long as supplies lasted [31]. To encourage continued testing, RAT kits were distributed to study participants starting in May 2023. Given the accessibility to testing throughout the study period, access and barriers to testing were not examined.

### 2.3. Data Collection

Following consent, participants were asked to complete a baseline questionnaire that captured demographic characteristics, health status and practices in the workplace, household and community that may be associated with the risk of respiratory infection. Vaccination self-reports collected the dates COVID-19 vaccines were received and the vaccine product name; they also included an option to indicate that no doses were (yet) received.

Participants were also asked to complete an illness report each time they were tested for SARS-CoV-2, regardless of the test result or type of test, as well as each time they had symptoms of a respiratory illness, even if they were not tested. Participants reported the dates of the symptom onset (as applicable), SARS-CoV-2 test type, test results, symptoms and exposures to people with COVID-19 prior to becoming symptomatic or being tested, as applicable. Participants had the opportunity to provide additional details as desired in an open-text question for general comments. For reports of symptomatic illness without a reported symptom onset date, the date of the SARS-CoV-2 test was considered as the symptom onset date (*n* = 5). In June 2021, the illness report was revised to reduce participant burden associated with daily reporting of ongoing symptomatic episodes; daily updates for the duration of symptoms were condensed into a single report.

### 2.4. Data Preparation

Due to the report structure, reporting of multiple tests within single episodes of illness was not likely to be valid because participants needed to either open a new report for the same illness or provide information in the comments section to report a second/subsequent test. As such, open-text responses in the illness reports were reviewed for additional details about reasons for testing and/or additional tests for which participants did not create individual reports. In instances of additional tests, new reports were created if sufficient detail was provided (i.e., date and reason for testing).

Responses to variables asking about symptoms, recent exposure to person(s) with COVID-19 and stated reason for testing were reviewed alongside open-text responses to categorise reason for testing into the following three categories: (1) symptomatic, with or without known or suspected COVID-19 exposure; (2) known or suspected exposure in the absence of symptoms and (3) routine testing, defined as testing with no reported symptoms and no known exposure (e.g., travel, social events, non–outbreak-related screening or fitness to work assessments). Testing ‘for symptoms' included any of the following symptoms: new or worsening cough, new shortness of breath, chest pain (pressure or heaviness), feeling feverish, chills or shivering, a fever of > 37.6° Celsius, feeling generally unwell, new onset of abnormal tiredness, confusion, new onset of generalised muscle aches or pains, new onset of joint pain, ear ache or infection, unusual headaches, sinus pain, sore or scratchy throat, new onset of a loss of appetite, nausea and/or vomiting, and diarrhoea and new onset of a loss in taste or smell. These liberal criteria were used because infection with SARS-CoV-2 has been associated with a wide range of symptoms [32, 33] and because testing of HCWs was initially recommended for the presence of any symptom [34].

An episode of symptomatic illness was defined as a report of new onset of any symptom(s) in a previously asymptomatic individual, starting from the reported symptom onset date. Reports with symptoms within a ±14-day window were considered one episode of symptomatic illness; this period was extended when a participant reported ongoing symptoms without resolution.

Participants were assigned the vaccination status they reported as of 10 days before the first day of each 4-week period (to account for time to mount an immune response). Categories were (1) never vaccinated or one dose of a two-dose vaccine received, (2) most recent dose (excluding first dose of a two dose primary series) received ≤ 6 months prior, (3) most recent dose (as above) received > 6 but ≤ 12 months prior and (4) most recent dose (as above) received > 12 months prior.

### 2.5. Statistical Analyses

#### 2.5.1. Rates of SARS-CoV-2 Testing

Outcomes were dichotomised (tested vs. not tested for SARS-CoV-2) for each 4-week period of participation. Rates of SARS-CoV-2 testing were calculated using 4-week periods starting on 16 June 2020, the date of the first submission of an illness report. Times at risk varied due to rolling enrolment, leaves of absence, changes of workplace and withdrawals. Denominator values (i.e., the total time at risk) were calculated for each 4-week period by summing the participants' times at risk during each period. Four-week periods were used to smooth the data and thereby more easily identify patterns by reducing the day-to-day noise in the data while minimising the bias that longer periods may introduce.

Calculations per 1000 person-days at risk were made for overall rates of testing as well as rates by reason for testing, province and vaccination status. To reduce the bias of repeat testing following negative initial reports, PCR ‘confirmatory' tests following a positive result by RAT, and return-to-work testing (to confirm negative results following infection), participant tests were eligible only once per 4-week period for the rates of overall, by province, and vaccination status testing. Rates of testing by reason for testing could include more than one test per participant per 4-week period but only if participants were tested for different reasons within the period. Reports with missing data on their reason for testing or vaccination status were excluded from the respective analyses.

Results are displayed alongside Canadian data of hospitalisation rates for COVID-19 [35] with a 9-day lag to incorporate time from symptom onset to hospitalisation [36]. Hospitalisation rates were used since they were less biased than case counts; case counts became increasingly unreliable after changes to eligibility for PCR testing in December 2021 [35].

#### 2.5.2. Time to First Test

Given the importance of rapid identification of infectious individuals to help reduce transmission, the time to first tests among HCWs with episodes of symptomatic illness was calculated using the median time between symptom onset and first reported test for SARS-CoV-2. The analysis was limited to tests conducted within 7 days of symptom onset since there is a steep decline in viral culture positivity after day five [37] that would reduce the benefit of being tested. Symptom onset was considered day zero, the succeeding day as day one and so forth.

#### 2.5.3. Retesting After Initial Negative Results

To investigate trends in retesting for SARS-CoV-2 for those who initially received a negative result, episodes of symptomatic illness were assigned to a 4-week period (as described above) based on symptom onset date. Episodes of symptomatic illness that were tested within 2 days of symptom onset and reported a negative result were reviewed for subsequent test(s) within 2 days of the negative test. The timing was based on the recommended timeline for retesting of 48 hours (when using RATs) by the U.S. Food and Drug Administration in 2022 [38] and by the Public Health Agency of Canada in 2023 [39].

Data editing and graphs were conducted using Microsoft Excel [40]. All analyses were conducted using Stata/SE 18.5 software [41].

## 3. Results and Discussion

**Figure 1:**
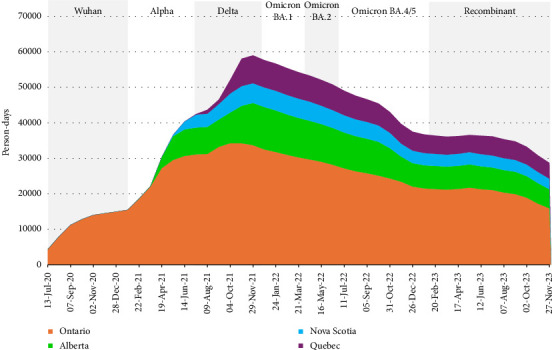
Person-days of participation by province and 4-week period; eligible Canadian healthcare workers in the COVID-19 Cohort Study, June 2020–November 2023.

Of the 2793 HCWs enrolled in the CCS, 2535 (90.8%) were eligible for inclusion in these analyses (81 did not complete a baseline report and 177 participated for < 30 days). The median age of those included was 40 years at enrolment (interquartile range [IQR] 32–51), and 2162 (85.3%) identified themselves as female, 1538 (60.7%) participants worked in Ontario. See Table 1 for complete demographic details. A timeline of recruitment and active participation of HCWs included in these analyses is provided in Figure 1. The median duration of participation was 690 days (IQR 358–926); the median number of illness episodes reported was 2 (IQR 1–3).

### 3.1. Rate of SARS-CoV-2 Testing

**Figure 2:**
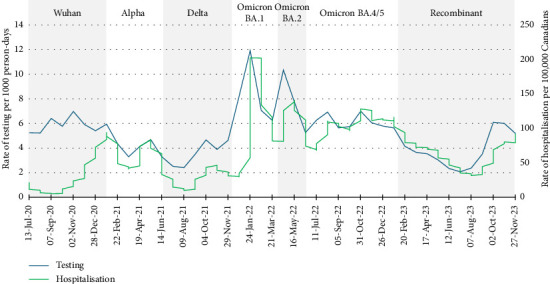
Rates of testing for SARS-CoV-2 by Canadian healthcare workers participating in the COVID-19 Cohort Study, June 2020–November 2023, and rates of hospital admission for COVID-19 in Canada, by 4-week periods. Blue: The rate of testing for SARS-CoV-2 per 1000 person-days within each 4-week period; green: weekly rate of hospitalisations for COVID-19 per 100,000 Canadians (all ages).

Rates of testing for SARS-CoV-2 fluctuated over the 3.5 years of data collection, as seen in Figure 2. Of note, the study began in June 2020, subsequent to increased access to testing for SARS-CoV-2 [7]. Rates of testing peaked at 11.9 participants tested at least once per 1000 person-days during the Omicron BA.1 wave, considerably higher than the 2.1 per 1000 person-days reported during the summer of 2023. Differences in rates may have been driven by changes in guidelines and policies, burden of community transmission of COVID-19 and other respiratory illnesses, as well as perceptions about individual susceptibility to SARS-CoV-2 and severity of COVID-19 [18, 42–44]. Supporting Figure S1 depicts the timing of some events that may have influenced testing behaviours such as the availability of RATs and vaccines, and vaccine mandates for HCWs.

**Figure 3:**
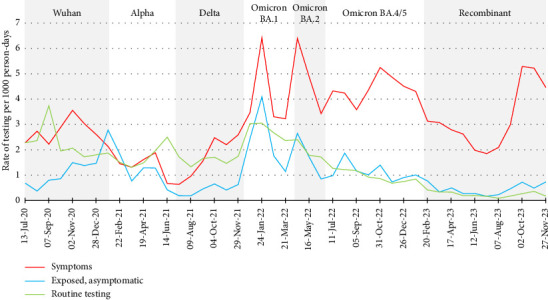
Rates of testing for SARS-CoV-2 by reason for testing, Canadian healthcare workers participating in the COVID-19 Cohort Study, June 2020–November 2023, by 4-week periods. Rates are per 1000 person-days, for each 4-week period. Eleven tests were excluded from this analysis due to missing reason for testing.

Using the rates of hospitalisation as a proxy for community transmission, the largest divergence between rates of HCW participant testing and rates of COVID-19-related hospitalisation occurred early in the study, from June to December 2020 (see Figure 2). As shown in Figure 3, rates of testing for routine purposes (no symptoms and no known exposures to people with COVID-19) were similar to rates of testing due to symptoms and testing due to exposure to a case until the start of the Omicron waves but declined in rank thereafter. This is likely because testing was used to enhance case finding and to reduce transmission of COVID-19 in healthcare settings prior to the availability of COVID-19 vaccines in Canada; vaccines were initially available in December 2020 but with very limited supply for several months [45, 46]. Routine testing of HCWs was promoted in 2020 as a tactic to reduce occupational spread from atypical, mild or asymptomatic cases of COVID-19 [47, 48]. Routine testing was implemented for international travel [49], staff and visitors in long-term care homes [50] or (infrequently) as an alternative to vaccination in settings with vaccine mandates [51].

A sharp increase in rates of testing coincided with the waves caused by the Omicron BA.1 and BA.2 variants in late 2021 through June 2022. As shown in Figure 3, participants reported that the reason for testing during this period was largely for symptoms compatible with COVID-19. The increase in the overall rate of testing (Figure 2) mirrors the increase in rates of hospital admission for COVID-19. This suggests that increasing community transmission does impact testing behaviours for people with symptoms. Similar to our results, Brankston et al. [52] reported that precautionary behaviours (avoiding close contact and indoor gatherings) among Canadian adults increased in the context of increasing COVID-19 incidence in 2020.

This period (late 2021/early 2022) also marked a dramatic shift in the eligibility for PCR testing, which was restricted to high-risk individuals and settings, along with increasing availability of RATs to the general public [44, 53, 54]. An Australian study found that increased test-seeking was associated with the increased availability of RATs [18]. Although RATs became the most commonly reported type of test used among those reporting positive results across Canada in January 2022 [54], a significant and sustained increase in testing was not noted in this study of HCWs. This may be because the HCW population remained eligible for PCR testing for many months after it was made more restrictive for the general public [9–12]. However, it may also be related, in part, to underreporting by study participants of testing with RATs, ostensibly those with negative results.

COVID-19 does not have a discernible seasonal pattern of infection [55], and although there is some variability in rates of testing by season, the patterns are not consistent. This was expected in 2020–2021 when nonpharmaceutical interventions to stop the spread of COVID-19 caused an ‘effective absence' of the annual seasonal respiratory virus epidemic in Canada [56]. A delayed return of influenza circulation within the community was observed in the spring of the 2021–2022 season in Canada coinciding with the easing of transmission-limiting interventions, with prepandemic-like influenza circulation patterns returning in the 2022–2023 season [57, 58]. Seasonal endemic cocirculation of multiple respiratory viruses in the winter months lends support for the utility of testing symptomatic HCWs for multiple pathogens using point-of-care multiplex tests in periods of high community transmission of respiratory pathogens [59]. In the meantime, rates of testing due to the presence of symptoms will likely be inflated during periods of cocirculating respiratory viruses making it necessary to consider these data in future analyses.

The reported rates of testing after exposure to someone with COVID-19—but prior to exhibiting symptoms—were generally lower than rates of testing due to being symptomatic (Figure 3). The rates of testing due to an exposure generally mirrored those for symptomatic testing (and hospitalisation) but steadily declined, relative to symptomatic testing, following the wave caused by the Omicron BA.1 variant. This may have been, in part, due to changes in testing recommendations for HCWs, and a need to reevaluate case and contact management procedures [60]. During 2020, enhanced case and contact management in Ontario included advising close contacts of cases to seek testing [61]. At the start of the Omicron BA.1 wave in December 2021, when increased community transmission and demand for testing outpaced the capacity of COVID-19 assessment centres [44], guidelines for testing were changed to include only symptomatic individuals with a higher risk of severe illness and/or symptomatic individuals working in high-risk settings, including acute care facilities [9–12].

**Figure 4:**
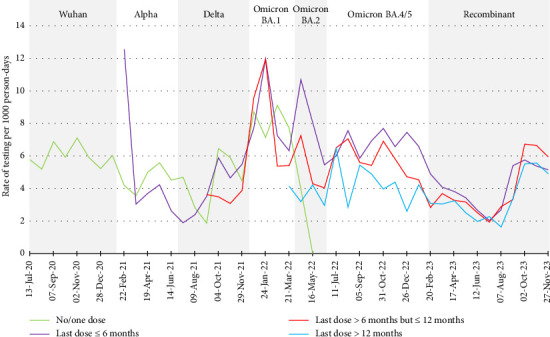
Rates of testing for SARS-CoV-2 by vaccination status, Canadian healthcare workers participating in the COVID-19 Cohort Study, June 2020–November 2023, by 4-week periods. Rates are per 1000 person-days, for each 4-week period. Seventy-six tests were excluded from this analysis due to missing vaccination status.

Overall rates of testing in this HCW population were generally lower in 2021 than in 2020. This may be attributed, in part, to increasing COVID-19 vaccination coverage [62]. A 2021 Health Canada report credited the reduction in test-seeking after the introduction of vaccines to a lower perception of risk from COVID-19, testing/pandemic fatigue and/or the social cost of a positive result outweighing the benefit of being tested at the individual level [63]. As shown in Figure 4, however, there was no discernible difference in rates of testing based on vaccination status in this sample of HCWs, with the notable exception of a spike in testing among recent recipients of vaccines in early 2021. This may reflect symptomatic testing due to vaccine side effects (that may have been hard to discern from SARS-CoV-2 symptoms). Policies implemented in the spring of 2021 in Ontario, where the majority of our participants resided, made it unnecessary to continue testing HCWs who had mild symptoms (headache, fatigue and myalgia/arthralgia *only*) within 48 hours after vaccination [64]. In contrast to our findings, Glasziou et al. [16] found that, in late 2021, fully vaccinated adult participants in Australia were twice as likely as unvaccinated ones to report intentions to be tested if they awoke with a sore throat. Alternatively, Kuitunen et al. [17], who surveyed the general public in Finland, found that those who had received three doses of vaccine against COVID-19 by January 2022 had the lowest testing rate, while those who were unvaccinated had the highest rate. These discrepant findings illustrate the need to understand each population's testing behaviours. Further, it has been suggested that symptomatic individuals may have attributed their symptoms to respiratory illnesses other than COVID-19 and therefore did not seek testing for this reason [42]; however, this last point was not stratified by vaccination status, which may be noteworthy given the change in symptom profile/burden associated with COVID-19 illness in vaccinated individuals [32, 65].

The social cost of a positive SARS-CoV-2 test result is difficult to interpret [63] and was also likely to have been affected by Canadian policies on paid sick leave during the COVID-19 pandemic. The (federal) Canada Recovery Sickness Benefit, that supported individuals without access to paid sick leave (including many part-time/casual HCWs) in the event they were sick or needed to self-isolate due to COVID-19, was in effect from September 2020 to May 2022 [66]. Provincial governments also implemented or modified existing paid sick leave policies, resulting in a variety of policies across the country [67] precluding us from making inferences about how employer-paid sick leave may have been associated with changes in testing behaviours in this cohort. Supporting Table S1 presents reasons for not being tested as volunteered by study participants. Of note, the reasons were not solicited so cannot be generalised to the entire cohort.

### 3.2. Rate of SARS-CoV-2 Testing by Province

**Figure 5:**
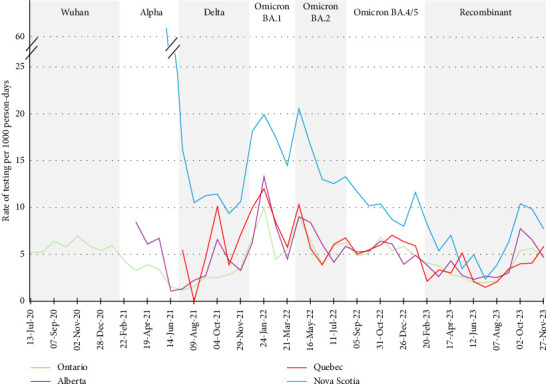
Rates of testing for SARS-CoV-2 by province, Canadian healthcare workers participating in the COVID-19 Cohort Study, June 2020–November 2023, by 4-week periods. Rates per 1000 person-days participation within each 4-week period.

HCWs in Ontario, Quebec and Alberta reported similar rates of testing, and although Nova Scotia followed similar trends in the timing of the peaks in rates of testing with the other three provinces, the rates were consistently higher (Figure 5). Although there are differences in population size, density and demographics between the provinces, there were also differences in their approach to dealing with the COVID-19 pandemic [68]. Such decisions are generally under the legislative responsibility of each of the provinces and territories in Canada [69]. During the pandemic, the Atlantic provinces (Nova Scotia, New Brunswick, Prince Edward Island, Newfoundland and Labrador) had a relatively successful, proactive and coordinated containment strategy, whereby they assertively traced and isolated cases and increased travel restrictions [68, 70]. Transmission rates remained low, and the Atlantic provinces were able to avoid province-wide lockdowns [68]. In comparison, Ontario, Quebec and Alberta experienced prolonged periods of lockdown and comparatively higher mortality rates [68]. Nova Scotia also implemented a ‘circuit breaker', a period of increased testing and restrictions in response to an increase in cases of COVID-19 in April 2021 [68]. This explains the initial peak in the rate of testing when Nova Scotian participants first enrolled in the study. Note that, despite attempts to smooth the rates by using 4-week periods, the relatively smaller number of participants in Alberta, Nova Scotia and Quebec compared with Ontario (see Figure 1) make the rates for those provinces more reactive to changes in testing that may have reflected outbreaks or periodic mass screening within the participating hospitals.

### 3.3. Time to First Test

Recalling that a symptomatic an illness was defined as illness with any of the above-listed symptoms, there were 5880 reports of symptomatic illness from 1933 participants. Of the episodes that were tested within seven days of symptom onset (*n* = 4984 or 84.8%), the median number of days between symptom onset and the first test was 1 (IQR 0–2); 29.5% of episodes were tested on the day of symptom onset and another 38.0% on the day following symptom onset.

Early in the pandemic, it was acknowledged that people working in healthcare settings may have faced an increased risk of exposure to SARS-CoV-2 given the likelihood of contact with individuals ill with COVID-19 [71] and that HCWs could initiate or amplify the spread of COVID-19 in healthcare settings if they worked while infectious [72, 73]. Our finding, that Canadian HCWs sought testing for SARS-CoV-2 upon recognition of symptoms, in about 30% of illness episodes, suggests they were cognizant of the risks associated with their employment and were responsive to actions intended to protect patients, coworkers and families. Similarly, Ga'al et al. [74] reported that 67% of HCWs in their study in Ontario, Canada, were tested within 3 days of the presentation of symptoms of COVID-19 between March 2020 and June 2021; HCWs had significantly higher odds (adjusted odds ratio 2.77; 95% confidence interval 2.52, 3.05) of testing within 3 days of symptom onset than non-HCWs. Within our study, 741 episodes (13%) received their first test three to seven days after symptom onset, and another 15% of episodes were not tested within seven days of symptom onset. These findings highlight that forward transmission was possible for many episodes of illness in this cohort of HCWs. Further study into the attitudes and risk factors associated with delayed or missed testing is warranted.

### 3.4. Retesting After Initial Negative Results

Testing too early or too late in the illness process may reduce test sensitivity [75]. This is further complicated by lower viral loads in vaccinated and previously infected individuals [76, 77] and a delay in peak viral loads with the Omicron variant [78]. As such, if people tested only once, and not at the optimal time, cases of COVID-19 may have been missed.

**Figure 6:**
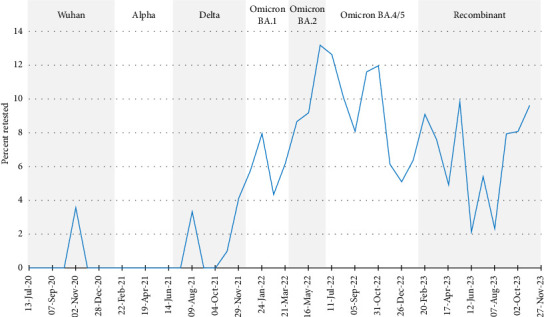
Rates of retesting for SARS-CoV-2 following an initial negative test result, Canadian healthcare workers participating in the COVID-19 Cohort Study, June 2020–November 2023, by 4-week periods. Percentage of participants within each 4-week period.

As shown in Figure 6, the overall rate of retesting after an initial negative test for symptomatic reports was low; it was < 14% throughout the duration of data collection. Low rates of retesting early in the pandemic were likely because repeat testing was not generally recommended when tested by PCR [9–12]. At that time, evidence suggested that repeat testing would not provide enough benefit to outweigh the burden it would have placed on the laboratory system, that was already overwhelmed [10]. It should be noted that it is also likely that study participants underreported repeat tests within one episode of illness due to the burden of doing so on the online reporting system (as noted in the data preparation section).

An increase in retesting following a negative test occurred towards the end of the Delta period, coinciding with the availability of RATs in Canada. While RATs improved access to testing, the difference in sensitivity and specificity of the tests must be considered. The U.S. Food and Drug Administration recommended repeat testing after 48 hours following a negative result on a RAT due to lower sensitivity compared with a PCR test [38, 79]. In addition, Frediani et al. [78] found low estimates of sensitivity of RATs on the first day of symptoms (30%–60%) from samples collected from symptomatic individuals, with sensitivity peaking on day four of symptoms (80%–93%), which is consistent with a delayed peak viral load observed during the Omicron and recombinant variant waves of the pandemic. It is important that these limitations are accounted for when interpreting surveillance, research and diagnostic data. It is also important to consider these limitations when using RATs to investigate infection with SARS-CoV-2 among HCWs. Best practices should include a second negative test result 48 hours after the first and, if the HCW must work, wearing a tight-fitting respirator or mask and modifying work duties to reduce transmission opportunities [80].

### 3.5. Study Strengths and Limitations

These data were collected over a 3.5 year period and four Canadian provinces enabling comparison across time and place, including the potential temporal influences of public policy and scientific advancements such as the introduction of the RAT and of COVID-19 vaccines. It enabled investigation of temporality, including whether symptom onset preceded SARS-CoV-2 testing, more sufficiently than many surveillance programs and retrospective studies. Further, our study captured multiple occupations within the healthcare environment that may acquire or transmit SARS-CoV-2 within their workplace, providing a more complete picture than those that only capture clinical roles. The online reporting platform and questionnaire also allowed for in-depth investigation into episodes of illness.

Given that information and events were self-reported, there was likely underreporting of illnesses and/or testing, as competing priorities related to responding to the COVID-19 pandemic would have made frequent reporting especially burdensome. Underreporting of negative tests was likely even more pronounced as was found in an analysis of public testing behaviours conducted in the United Kingdom [81]. Further, the reporting structure precluded authors from accurately deciphering between a test reported twice, in error, and multiple tests occurring on the same day. Therefore, and to reduce the effect of under- or overreporting, testing within each 4-week period was used for these analyses. Social desirability bias may have also contributed to underreporting as it was possible that symptomatic participants who were not tested did not submit an illness report. The use of anonymised surveys was used in an attempt to reduce these biases; however, it was not possible to reduce the burden of completing reports.

This study relied on voluntary participation making it unlikely that participants were representative of all HCWs in Canada. In addition, other studies report selection bias as health-conscious individuals were more likely to participate in studies [82] and that may inflate the observed rates of testing for SARS-CoV-2 among HCWs. The dynamic nature of participation in the CCS also increases the possibility of differences among participants over time that could potentially bias rates of testing if participants who were more or less likely to test were also more or less likely to continue their participation in the study. The rolling enrolment by province may reduce the generalisability of our findings in 2020 and early 2021, before all four participating provinces were enrolled. Finally, participating hospitals were located in large metropolitan areas so findings may not be generalisable to healthcare facilities in smaller jurisdictions.

## 4. Conclusions

Testing for SARS-CoV-2 within the Canadian HCW study population fluctuated over the 3.5 years of data collection with rates generally following the rates of hospitalisation for COVID-19 in Canada, a proxy for rates of infection. The spread of the Omicron variant signalled a shift in the way Canadian governments dealt with the COVID-19 pandemic, and there was a shift in the patterns of testing at this point. As such, monitoring and understanding the effects of internal (e.g., HCWs ability to recognise COVID-19 symptoms, attitudes towards testing or perceived risks of testing) and external (e.g., testing eligibility and availability) influences on testing rates and behaviours over time is important for interpreting data for vaccine effectiveness studies and surveillance information that impacts public health decision-making. It can also highlight instances of missed case–finding opportunities that can drive transmission in healthcare settings.

## Figures and Tables

**Table 1:** Characteristics of eligible Canadian healthcare workers at enrolment; COVID-19 Cohort Study (June 2020–November 2023), number (percent) unless otherwise noted.

Characteristic	Participants (*N* = 2535)
*Age in years,* median (IQR)	40 (32–51)

*Gender*	
Female	2162 (85.3)
Male	364 (14.4)
Other	9 (0.4)

*Occupation*	
Nurse/nurse practitioner/midwife	833 (32.9)
Physician/physician assistant	273 (10.8)
Other regulated health worker^1^	741 (29.2)
Other^2^	688 (27.1)

*Province*	
Ontario	1538 (60.7)
Alberta	460 (18.1)
Nova Scotia	226 (8.9)
Quebec	311 (12.3)

Abbreviation: IQR, interquartile range.

^1^Respiratory therapist, laboratory technician, physical therapist, occupational therapist, imaging technician/technologist, pharmacist, pharmacy technician, psychologist and social worker.

^2^Food service, ward clerk, administration, healthcare aids, housekeeper, porter, research and other clinical support.

## Data Availability

The data that support the findings of this study are available on request from the corresponding author. The data are not publicly available due to privacy or ethical restrictions.

## Supporting Information

Supporting Information: Trends in testing for SARS-CoV-2 among healthcare workers in a Canadian Cohort Study during the COVID-19 pandemic, June 2020 to November 2023.

Supporting Figure 1: Timeline of notable events during the COVID-19 Cohort Study data collection period.

Supporting Table S1: Reasons for not being tested for SARS-CoV-2 volunteered by study participants of the COVID-19 Cohort Study, June 2020–November 2023.

Supporting Table S2: Completed STROBE Statement; SARS-CoV-2 testing trends among healthcare workers in the COVID-19 Cohort Study, June 2020–November 2023.
