# Supplementary material for: Trends in Testing for SARS-CoV-2 Among Healthcare Workers in a Canadian Cohort Study During the COVID-19 Pandemic, June 2020 to November 2023
Source: Can J Infect Dis Med Microbiol. 2025 May 30;2025:1858884. doi: 10.1155/cjid/1858884 (PMC12143943; doi:10.1155/cjid/1858884)
Supplement: Supporting Information — Additional supporting information can be found online in the Supporting Information section. [file 1858884.f1.pdf]

Supplementary Material for Trends in testing for SARS-CoV-2 among healthcare workers in a Canadian cohort study during the COVID-19 pandemic, June 2020 to November 2023

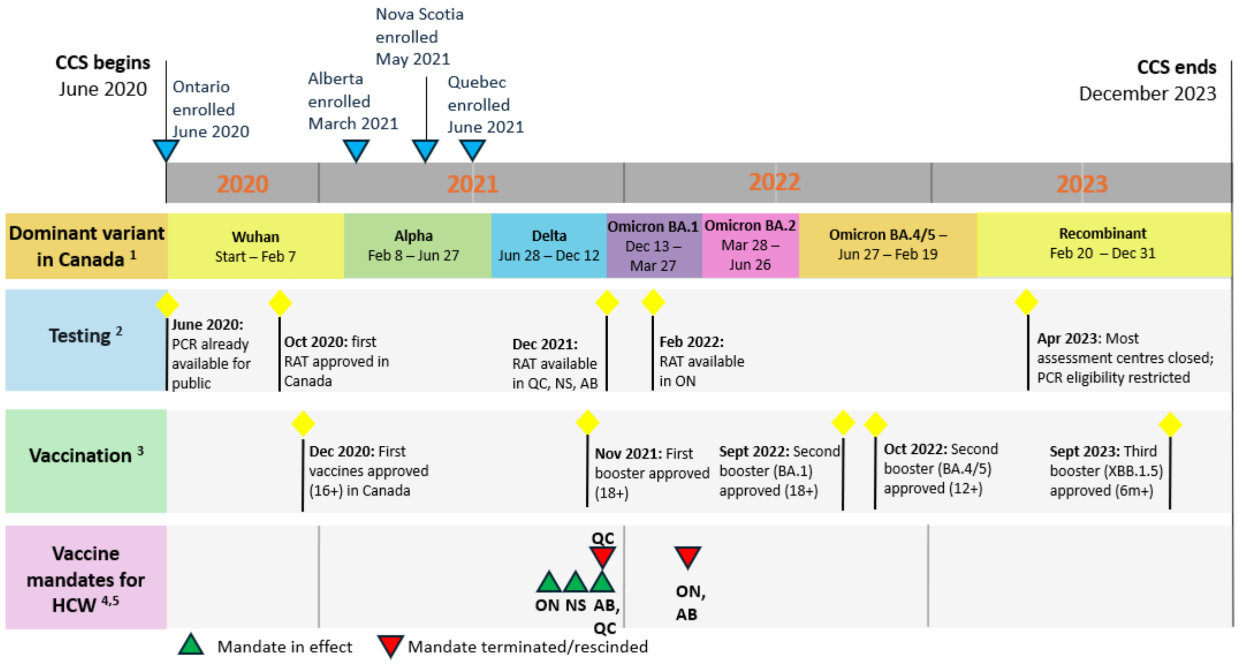

Supplementary Figure 1: Timeline of notable events during the COVID-19 Cohort Study data collection period

CCS: COVID-19 Cohort Study; ON: Ontario; QC: Quebec; NS: Nova Scotia; AB: Alberta
PCR: polymerase chain reaction test; RAT: rapid antigen test; HCW: healthcare worker

References for Figure S1:

[1] Dominant variant in Canada: Government of Canada. COVID-19 epidemiology update: Current situation [Internet]. 2024 [cited 2024 Mar 5]. Available from: https://health-infobase.canada.ca/covid-19/current-situation.html.

[2] Testing: Ontario Ministry of Health. COVID-19 Provincial Testing Guidance Update: April 15, 2020 [Internet]. 2020 [cited 2024 Sept 30]. Available from: https://www.corhealthontario.ca/Item-4-Provincial-Testing-Guidance-Update.pdf.;
Health Canada. Pan-Canadian COVID-19 Testing and Screening Guidance: Technical guidance and implementation plan [Internet]. 2021 [cited 2024 Oct 1]. Available from: https://www.canada.ca/en/health-canada/services/drugs-health-products/covid19-industry/medical-devices/testing/pan-canadian-guidance.html.;
Alberta Health. COVID-19 information : COVID-19 rapid testing : information sheet [Internet]. 2021 [cited 2024 Sept 15]. Available from: https://open.alberta.ca/publications/covid-19-information-covid-19-rapid-testing#detailed.;
Government of Nova Scotia. Rapid Tests Available at Libraries for Holiday Season [Internet]. 2021 [cited 2024 Sept 2]. Available from: https://news.novascotia.ca/en/2021/12/13/rapid-tests-available-libraries-holiday-season.;

Ministère de la Santé et des Services sociaux Québec. Pandémie de la COVID-19 - Des tests de dépistage rapide distribués graduellement pour l’ensemble de la population [Internet]. 2021 [cited 2024 Sept 15]. Available from: https://www.msss.gouv.qc.ca/ministere/salle-de-presse/communique-3350/.;
Government of Ontario. Rapid testing for at-home use [Internet]. 2022 [cited 2024 Sept 15]. Available from: https://www.ontario.ca/page/rapid-testing-home-use.;
Government of Ontario. Ontario Expanding Access to Free Rapid Tests for General Public [Internet]. 2022 [cited 2024 Sept 15]. Available from: https://news.ontario.ca/en/release/1001575/ontario-expanding-access-to-free-rapid-tests-for-general-public.;
Government of Ontario. COVID-19 testing and treatment [Internet]. 2022 [cited 2024 Sept 15]. Available from: https://www.ontario.ca/page/covid-19-testing-and-treatment.;
Government of Québec. Getting a COVID-19 test [Internet]. 2023 [cited 2024 Oct 1]. Available from: https://web.archive.org/web/20230202174514/https://www.quebec.ca/en/health/advice-and-prevention/screening-and-carrier-testing-offer/testing-flu-like-symptoms/get-covid-19-test#c148948. ;
Nova Scotia Health. COVID-19 Testing [Internet]. 2023 [cited 2025 Oct 1]. Available from: https://web.archive.org/web/20230406001955/https://www.nshealth.ca/coronavirustesting.;
Government of Ontario. COVID-19 testing and treatment [Internet]. 2023 [cited 2024 Oct 1]. Available from: https://web.archive.org/web/20231202030325/https://www.ontario.ca/page/covid-19-testing-and-treatment.

[3] Vaccines: Health Canada. Health Canada authorizes first COVID-19 vaccine [Internet]. 2020 [cited 2024 Oct 1]. Available from: https://www.canada.ca/en/health-canada/news/2020/12/health-canada-authorizes-first-covid-19-vaccine0.html.;
Health Canada. Health Canada authorizes the use of the Pfizer-BioNTech Comirnaty COVID-19 vaccine as a booster shot [Internet]. 2021 [cited 2024 Oct 1]. Available from: https://www.canada.ca/en/health-canada/news/2021/11/health-canada-authorizes-the-use-of-the-pfizer-biontech-comirnaty-covid-19-vaccine-as-a-booster-shot.html.;
Health Canada. Health Canada authorizes first bivalent COVID-19 booster for adults 18 years and older [Internet]. Government of Canada; 2022 [cited 2024 Oct 1]. Available from: https://www.canada.ca/en/health-canada/news/2022/09/health-canada-authorizes-first-bivalent-covid-19-booster-for-adults-18-years-and-older.html.;
Health Canada. Health Canada authorizes COVID-19 vaccine booster targeting the Omicron BA.4/BA.5 subvariants [Internet]. 2022 [cited 2024 Oct 1]. Available from: https://www.canada.ca/en/health-canada/news/2022/10/health-canada-authorizes-covid-19-vaccine-booster-targeting-the-omicron-ba4ba5-subvariants.html.;
Health Canada. Health Canada authorizes Moderna COVID-19 vaccine targeting the Omicron XBB.1.5 subvariant [Internet]. 2023 [cited 2024 Oct 1]. Available from: https://www.canada.ca/en/health-canada/news/2023/09/health-canada-authorizes-moderna-covid-19-vaccine-targeting-the-omicron-xbb15-subvariant.html.

[4] Mandates: Government of Ontario.
Ontario Makes COVID-19 Vaccination Policies Mandatory for High-Risk Settings [Internet]. 2021 [cited 2024 Oct 1]. Available from: https://news.ontario.ca/en/release/1000750/ontario-makes-covid-19-vaccination-policies-mandatory-for-high-risk-settings.;
Government of Nova Scotia. Vaccination Required for Provincial Government Employees [Internet]. 2021 [cited 2024 Oct 1]. Available from: https://news.novascotia.ca/en/2021/10/06/vaccination-required-provincial-government-employees.;
Government of Alberta. New vaccination policy for Alberta Public Servants [Internet]. 2021 [cited 2024 Sept 15]. Available from: https://www.alberta.ca/release.cfm?xID=79917372CAAE4-A799-9E9B-C3F39550E2141819.;

Ministry of Health and Social Services Quebec. Quebec government confirms mandatory vaccination on October 15 [Internet]. 2021 [cited 2024 Sept 15]. Available from: https://www.msss.gouv.qc.ca/ministere/salle-de-presse/communique-3171/.;
Ministry of Health and Social Services Quebec. To ensure continuity of services - Mandatory vaccination postponed to November 15 [Internet]. 2021 [cited 2024 Sept 15]. Available from: https://www.msss.gouv.qc.ca/ministere/salle-de-presse/communique-3204/.;
Ministry of Health and Social Services Quebec. To maintain essential services - Health and social services workers who are not adequately protected will be able to continue working under several conditions [Internet]. 2021 [cited 2024 Sept 15]. Available from: https://www.msss.gouv.qc.ca/ministere/salle-de-presse/communique-3252/.;
Government of Ontario. Minister's Directive: Long-term care home COVID-19 immunization policy [Internet]. 2022 [cited 2024 Oct 1]. Available from: https://www.ontario.ca/page/ministers-directive-long-term-care-home-covid-19-immunization-policy.;
Government of Alberta. AHS mandatory vaccination policy lifted [Internet]. 2022 [2024]. Available from: https://www.alberta.ca/release.cfm?xID=8206835279AEF-F976-3D2F-EAA53CFCDD239C52.;

[5] Nova Scotia continued to require COVID-19 vaccination as a condition of employment for healthcare workers; this was rescinded on February 26, 2024; Nova Scotia Health Authority, Health I. Joint position statement: Amendment to COVID-19 vaccination policy [Internet]. 2024 [cited 2024 Oct 21]. Available from: https://policy.nshealth.ca/Site_Published/covid19/document_render.aspx?documentRender.IdType=6&documentRender.GenericField=&documentRender.Id=106648#:~:text=Based%20on%20the%20current%20available,primary%20series%20COVID%2D19%20immunization.

Supplementary Table S1 Reasons for not being tested for SARS-CoV-2 volunteered by study participants of the COVID-19 Cohort Study, June 2020-November 2023

*Note that these reasons were selected and presented only to illustrate the experiences of participants; formal qualitative analyses were not undertaken so cannot be generalized to the entire cohort

Reason	Quotes
Attributing symptoms to post-vaccination side effects	*“I'm pretty sure the symptoms I experienced were merely a side effect of the booster shot.”*
	“Diarrhea, vomiting, loss of smell...all side effects of the booster”
Receiving an alternative diagnosis/were told not to be tested by healthcare professional	*“Was diagnosed with strep throat”*
	“Felt overly fatigued for 5 days - missed one day of work. Was not required to get Covid tested”
	“Occupational Health did not recommend I get swabbed for COVID”
Attributing their symptoms to another cause	*“My son got flu from daycare and we all got it”*
	“I have a cold. (No fever, chills) I just need a good night's sleep”
	“My children had been tested the week before due to cold symptoms and they were negative”
	“Coughing due to smoky conditions from forest fires”
Attributing symptoms to a previous SARSCoV-2 infection	*“Still lingering symptoms from my covid infection - seems to get better and then worse and back and forth”*
Not having access to COVID-19 tests	*“During holidays. I had no COVID test with me”*
Assuming positive due to exposure	*“Only work from home so did not PCR. Spouse was positive on RAT”*

Supplementary Table 2 STROBE Statement for SARS-CoV-2 testing trends among healthcare workers in the COVID-19 Cohort Study, June 2020-November 2023

	Item No	Recommendation	Completed?
**Title and abstract**	1	(*a*) Indicate the study's design with a commonly used term in the title or the abstract	Yes
		(*b*) Provide in the abstract an informative and balanced summary of what was done and what was found	Yes
**Introduction**			
Background/rationale	2	Explain the scientific background and rationale for the investigation being reported	Yes
Objectives	3	State specific objectives, including any prespecified hypotheses	Yes
**Methods**			
Study design	4	Present key elements of study design early in the paper	Yes
Setting	5	Describe the setting, locations, and relevant dates, including periods of recruitment, exposure, follow-up, and data collection	Yes
Participants	6	(*a*) Give the eligibility criteria, and the sources and methods of selection of participants. Describe methods of follow-up	Yes
		(*b*) For matched studies, give matching criteria and number of exposed and unexposed	N/A
Variables	7	Clearly define all outcomes, exposures, predictors, potential confounders, and effect modifiers. Give diagnostic criteria, if applicable	Yes
Data sources/ measurement	8*	For each variable of interest, give sources of data and details of methods of assessment (measurement). Describe comparability of assessment methods if there is more than one group	Yes
Bias	9	Describe any efforts to address potential sources of bias	Yes
Study size	10	Explain how the study size was arrived at	N/A
Quantitative variables	11	Explain how quantitative variables were handled in the analyses. If applicable, describe which groupings were chosen and why	Yes
Statistical methods	12	(*a*) Describe all statistical methods, including those used to control for confounding	Yes
		(*b*) Describe any methods used to examine subgroups and interactions	Yes
		(*c*) Explain how missing data were addressed	Yes
		(*d*) If applicable, explain how loss to follow-up was addressed	N/A
		(*e*) Describe any sensitivity analyses	N/A
**Results**			
Participants	13*	(a) Report numbers of individuals at each stage of study—eg numbers potentially eligible, examined for eligibility, confirmed eligible, included in the study, completing follow-up, and analysed	Yes
		(b) Give reasons for non-participation at each stage	Yes
		(c) Consider use of a flow diagram	N/A

Descriptive data	14*	(a) Give characteristics of study participants (eg demographic, clinical, social) and information on exposures and potential confounders	Yes
		(b) Indicate number of participants with missing data for each variable of interest	Yes
		(c) Summarise follow-up time (eg, average and total amount)	Yes
Outcome data	15*	Report numbers of outcome events or summary measures over time	Yes
Main results	16	(*a*) Give unadjusted estimates and, if applicable, confounder-adjusted estimates and their precision (eg, 95% confidence interval). Make clear which confounders were adjusted for and why they were included	N/A
		(*b*) Report category boundaries when continuous variables were categorized	Yes
		(*c*) If relevant, consider translating estimates of relative risk into absolute risk for a meaningful time period	N/A
Other analyses	17	Report other analyses done—eg analyses of subgroups and interactions, and sensitivity analyses	Yes
**Discussion**			
Key results	18	Summarise key results with reference to study objectives	Yes
Limitations	19	Discuss limitations of the study, taking into account sources of potential bias or imprecision. Discuss both direction and magnitude of any potential bias	Yes
Interpretation	20	Give a cautious overall interpretation of results considering objectives, limitations, multiplicity of analyses, results from similar studies, and other relevant evidence	Yes
Generalisability	21	Discuss the generalisability (external validity) of the study results	Yes
**Other information**			
Funding	22	Give the source of funding and the role of the funders for the present study and, if applicable, for the original study on which the present article is based	Yes

References for Table S2:

von Elm E, Altman DG, Egger M, Pocock SJ, Gøtzsche PC, Vandenbroucke JP. The Strengthening the Reporting of Observational Studies in Epidemiology (STROBE) statement: guidelines for reporting observational studies. J Clin Epidemiol. 2008;61(4):344-9. doi:10.1016/j.jclinepi.2007.11.008
